# Is Long-Term Exposure to Ambient Air Pollution a Risk Factor for Parkinson’s Disease.

**DOI:** 10.23889/ijpds.v7i3.1822

**Published:** 2022-08-25

**Authors:** Babak Jahanshahi, Duncan McVicar, Neil Rowland, Mark McGovern, Dermot O'Reilly

**Affiliations:** 1 Queen's Management School, Queen's University Belfast; 2 Rutgers School of Public Health; 3 Centre of Excellence for Public Health, Queen’s University Belfast

## Objectives

This paper links prescriptions data for the Northern Ireland population with data from the Northern Ireland Longitudinal Study and localized ambient air pollution data from 2002 onwards to estimate the association between long-term exposure to ambient air pollutant from fine particulates (PM2.5) and Parkinson’s Disease (PD).

## Approach

Cox Proportional Hazards models are used to examine the impact of air pollution on PD, first unconditionally, and then conditioning on a rich set of observable individual, family and contextual characteristics. Long-term exposure to PM2.5 is defined as exposure averaged over the previous 5 years. Onset of PD is proxied by first receipt of a prescription for PD medication. Estimates are presented in the form of hazard ratios for the effect of long-term PM2.5 exposure on the risk of PD onset.

## Results

There is a non-trivial magnitude and statistically significant unconditional association between long-term exposure to ambient PM2.5 pollution and receiving a prescription for PD, with those exposed to higher levels of pollution more likely to receive a prescription for PD. This estimated association disappears (becomes insignificantly different from zero), however, when the model accounts for confounding variables at household, individual and geographical levels.

## Conclusion

This study contributes to an emerging literature examining the association between ambient PM2.5 pollution and onset of PD. Despite finding an unconditional association, we find no evidence for an association once individual, family and contextual characteristics are controlled for, at least in the relatively low-pollution context of Northern Ireland.

